# IRES-dependent translated genes in fungi: computational prediction, phylogenetic conservation and functional association

**DOI:** 10.1186/s12864-015-2266-x

**Published:** 2015-12-15

**Authors:** Esteban Peguero-Sanchez, Liliana Pardo-Lopez, Enrique Merino

**Affiliations:** Departamento de Microbiología Molecular, Instituto de Biotecnología, UNAM, Av. Universidad 2001, Cuernavaca, Morelos CP 62210 Mexico

**Keywords:** IRES, mRNA, Translation, Comparative genomics, Fungi, SVM, Protein-protein interaction networks, Stress response

## Abstract

**Background:**

The initiation of translation via cellular internal ribosome entry sites plays an important role in the stress response and certain physiological conditions in which canonical cap-dependent translation initiation is compromised. Currently, only a limited number of these regulatory elements have been experimentally identified. Notably, cellular internal ribosome entry sites lack conservation of both the primary sequence and mRNA secondary structure, rendering their identification difficult. Despite their biological importance, the currently available computational strategies to predict them have had limited success. We developed a bioinformatic method based on a support vector machine for the prediction of internal ribosome entry sites in fungi using the 5’-UTR sequences of 20 non-redundant fungal organisms. Additionally, we performed a comparative analysis and characterization of the functional relationships among the gene products predicted to be translated by this cap-independent mechanism.

**Results:**

Using our method, we predicted 6,532 internal ribosome entry sites in 20 non-redundant fungal organisms. Some orthologous groups were enriched with our positive predictions. This is the case of the HSP70 chaperone family, which remarkably has two verified internal ribosome entry sites, one in humans and the other in flies. A second example is the orthologous group of the eIF4G repression protein Sbp1p, which has two homologous genes known to be translated by this cap-independent mechanism, one in mice and the other in yeast. These examples emphasize the wide conservation of these regulatory elements as a result of selective pressure. In addition, we performed a protein-protein interaction network characterization of the gene products of our positive predictions using *Saccharomyces cerevisiae* as a model, which revealed a highly connected and modular topology, suggesting a functional association. A remarkable example of this functional association is our prediction of internal ribosome entry sites elements in three components of the RNA polymerase II mediator complex.

**Conclusions:**

We developed a method for the prediction of cellular internal ribosome entry sites that may guide experimental and bioinformatic analyses to increase our understanding of protein translation regulation. Our analysis suggests that fungi show evolutionary conservation and functional association of proteins translated by this cap-independent mechanism.

**Electronic supplementary material:**

The online version of this article (doi:10.1186/s12864-015-2266-x) contains supplementary material, which is available to authorized users.

## Background

Eukaryotic cells regulate the synthesis of proteins using various mechanisms. Among them, protein translation control provides faster changes in protein levels when compared, for example, to transcriptional responses [1]. Under stress and other physiological and physiopathological conditions, translation is heavily repressed to conserve cellular resources. Nevertheless, a set of proteins, mostly related to stress responses that mediate cell adaptation to diverse stimuli or that are necessary for the regulation of developmental processes, are selectively synthesized. The prevalence of translational control has been assessed in yeast and other fungal organisms [2–5]. One of the mechanisms that allows such selective protein expression under these conditions is internal ribosome entry site (IRES)-dependent translation [1, 6–9].

Importantly, translation initiation is widely considered to be the most regulated step in protein translation [1]. Under normal conditions, translation initiation proceeds via the canonical or 5’-cap-dependent mechanism. In this process, the translation machinery recognizes the 5’-m^7^G-cap modification of the mRNA, paving the way for translation initiation. However, under certain circumstances, some components of the translation machinery are depleted, and 5’-cap recognition is suppressed. These conditions hinder the canonical translation initiation mechanism. IRESs allow the binding of the translation machinery to mRNA independently of 5’-cap recognition, enabling translational initiation to proceed [6, 7, 10]. The first IRES was reported in the 5’-UTR of picornaviruses [11]. Subsequently, a number of IRESs were described in multiple viral transcripts. They enable viral protein production using the host translational machinery when the global synthesis is repressed due to the infection process. Shortly afterwards, the first cellular IRES was identified in the 5’-UTR of the BiP chaperone, allowing its translation in poliovirus-infected cells [12]. Currently, there are more than 100 reported cellular IRESs [13].

Until now, a high-throughput method for the detection of IRESs is not available; each candidate has to be tested individually in a procedure that involves different stringent controls to verify its activity [14]. Thus, we believed that a bioinformatic approach to discover new potential IRESs would vastly reduce the number of candidates to be tested. Nevertheless, the prediction of cellular IRESs presents a considerable challenge due to their lack of sequence and structure conservation, even in homologous genes [15]. For this reason, and to the best of our knowledge, current predictive strategies have had very limited success [16]. To develop a computational methodology to identify IRES-specific patterns and accurately predict these regulatory elements in fungal species, we implemented a support vector machine (SVM) method using 5’-UTR sequence characteristics and comparative genomic features. Subsequently, an enrichment analysis of our initial IRES predictions in clusters of orthologous yeast genes allowed us to identify the most likely IRES candidates. In this article, using *S. cerevisiae* as a model, we present a detailed analysis of the protein-protein interaction (PPI) network of genes translated by these top IRES predictions. The notable enrichment in orthologous groups, the PPI analysis and the functional evaluation of our predictions enabled us to formulate biological hypotheses concerning the evolutionary conservation and genome-wide associations of IRESs.

## Results and discussion

### Prediction of IRESs in 5’-UTR regions

To identify 5’-UTR regions bearing IRESs, we developed a method based on machine learning and comparative genomics. Our method is focused on the unstructured A-rich IRESs found in fungal organisms, such as those identified in *S. cerevisiae* [17, 18], and does not include highly structured IRESs found in higher organisms [16]. We employed sequence composition-based features, the minimum folding energies (MFEs) of the RNAs, and certain orthologous group comparative properties to generate a total of 29 features, which are listed in Additional file 1 (see Methods).

Cross-validation was performed to evaluate the performance of our method and for parameter optimization. The SMOTE procedure allowed the generation of synthetic positive cases that were used for training and testing, as described in the Methodology section. This set (consisting of positive and negative cases) was randomly split into ten parts; of these, one was used for testing, and the rest were used for training. The process continued until all the parts were individually used for training (10 steps). The performance measures were determined (accuracy and Cohen’s kappa) for each of the steps, and the mean values were calculated. This entire process was repeated 30 times. From a total of 32 different combinations of SVM parameters, the optimized parameters were sigma and cost. We selected the model with the best performance measures achieving an accuracy of 94.3 % and a Cohen’s kappa of 0.828 [19, 20]. The estimated values of sensitivity and specificity using a confusion matrix for our model were 0.94 and 0.98, respectively. Thereafter, we used our model to make predictions for 99,759 sequences from 20 independent fungal organisms. Our method classified 6,532 sequences as containing IRESs (positive predictions) and 93,227 sequences as not containing IRESs (negative predictions). The positive predictions represent 6.8 % of the total sequences used. This number is in close agreement with the estimate of the proportion of cellular mRNAs that could be translated using cap-independent mechanisms, according to cDNA microarray data (10-15 %) [21, 22]. In order to have a negative control with the exactly the same number of sequences as in our IRES analysis, for every gene initially considered in our study, we analyzed the 60 nt immediately upstream of the translation termination codon since it is expected that in this coding region, the presence of IRES would be minimal or nonexistent. The number of sequences analyzed as negative control was 99,759 and of these only 317 were predicted as containing IRESs (false positives). This corresponds to 0.3 % of the total negative control set. Considering that our IRES analysis included the same number of UTR sequences (99,759) and that 6,532 of them were predicted as containing IRESs, the false discovery rate of our predictions was evaluated to be 5 %.

### Evolutionarily conserved patterns related to IRES-dependent translation are found in the 5’-UTRs of fungi

**Fig. 1:**
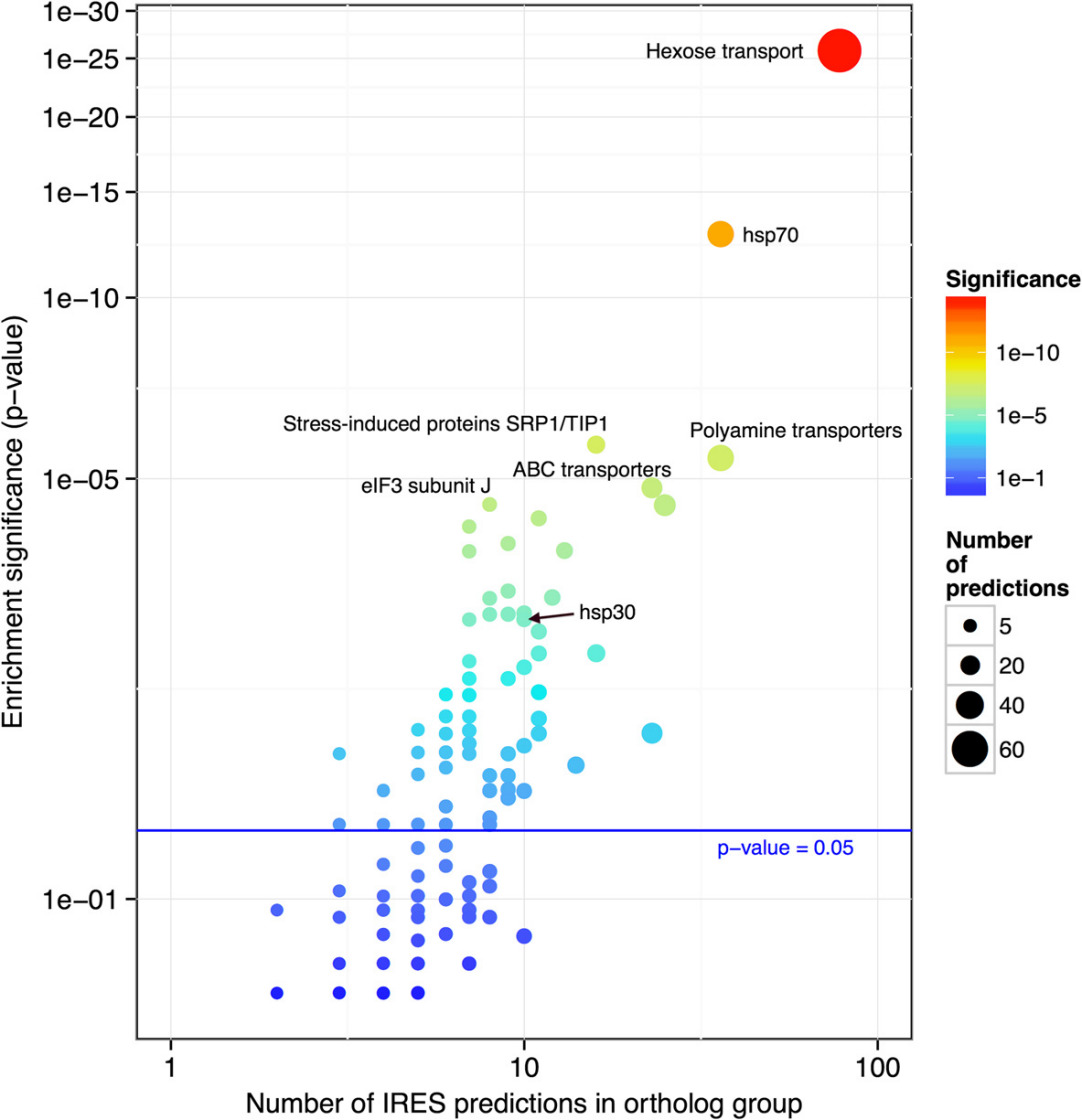
Predictions of enriched orthologous groups. Some of the most relevant groups are labeled. The statistical significance of the enriched orthologous groups is highlighted. The number of predictions within each group is proportional to the size of the points

Of the aforementioned 6,532 positive predictions, 815 showed distinctive features of evolutionary conservation as analyzed by orthologous group enrichment (False discovery rate (FDR) < 0.05). These predictions included 86 orthologous groups (OG) out of the 22,605 considered (Fig. 1).

Several of the enriched groups contain genes implicated in the stress response. Some of these groups have homologous genes experimentally verified as genes containing IRESs in other organisms. We discuss the biological relevance of the most enriched groups below.

The most significant group yielded an enrichment *p*-value of 1 x 10^−26^, corresponding to 78 positive predictions out of the 265 gene members of this group encoded in the 20 non-redundant fungal genomes used in our analysis. The proteins in this group are transmembrane sugar transporters and glucose sensors (hexose transporters group). These transporters have a wide array of affinities and are regulated by glucose concentration, allowing adaptation to changing conditions in nutrient levels; their function and regulation are reviewed in [23]. Furthermore, experiments using ribosome profiling analysis have shown that six hexose transporters genes—*HXT1*, *HXT2*, *HXT4*, *HXT5*, *HXT9*, and *GAL2—*are translationally up-regulated in response to osmotic stress [24]. Importantly, the genes encoding four of these proteins—Hxt1p, Hxt5p, Hxt9p and Gal2p—were predicted to contain IRESs using our method in most of our studied organisms. Translational up-regulation was preferentially mediated by strengthened polysome association in the 5’-UTR after osmotic stress and not only by increased mRNA levels. Additionally, an increase in polysomal mRNA led to incremental protein production [24]. This finding is in good agreement with our predictions because increased ribosome occupancy in the 5’-UTR has been linked to IRES-dependent translation [3].

The second-most enriched group corresponds to the HSP70 family (36 predictions out of 107, *p*-value of 1.7 x 10^−13^). Acting as chaperones, the proteins in this family are conserved in virtually all organisms and are used by cells to contend with several types of stress, including heat. There is evidence of translational control and increased ribosome occupancy in the mRNA of *SSA4* (which was predicted to contain an IRES by our model) in response to different stress conditions, such as high salinity [25] and starvation conditions, in which its translation efficiency increased 2.5-fold [2]. There are two members of this family that have experimentally verified IRESs, one in humans and the other in flies [13]. This result may be explained by the hypothesis that IRES-dependent translation initiation is conserved across species in phylogenetically related proteins.

The third-most enriched group includes the stress-induced Srp1p/Tip1p family (16 predictions out of 40, *p*-value of 2.0 x 10^−6^). Several members of this family are known to be induced by various stress conditions, including low temperatures [26], hypoxia [27] and nitrogen starvation [28]. A number of members of the SRP/TIP1 family are regulated by the transcriptional factor Mss11p [29]. Remarkably, Mss11p, Msn1p and Flo8p are part of the signal transduction pathway that regulates pseudohyphal differentiation and filamentous growth [30]. Furthermore, Mss11p and Flo8p bind cooperatively to the *STA1* promoter, leading to the filamentous and invasive growth response [31]. Significantly, the genes coding for Flo8p and Msn1p are translated in an IRES-dependent manner, as are 7 additional genes involved in invasive growth [17]. One hypothesis that could explain these observations is that IRES-dependent translation is required when the selective co-expression of proteins under stress conditions is needed, for example, in some regulatory or interaction networks.

The fourth-most enriched group (36 predictions out of 185, *p*-value of 3.9 x 10^−6^) represents a subset of the major facilitator superfamily, more specifically genes that code for H^+^ antiporters. These enzymes are crucial for multidrug resistance and chemical stress responses in yeast [32]. In this regard, it has been demonstrated that *PDR15* is translationally regulated and that its 5’-UTR shows increased levels of ribosome occupancy in response to high salinity [25]. Similarly, *PDR5* and *PDR12* (which were positive IRES predictions according to our model) showed a positive correlation between ribosome 5’-UTR occupancy and translational efficiency during different developmental stages; this trend has been linked to translationally regulated genes. Importantly, a similar correlation was observed for yeast IRESs [3].

The fifth-most significant group (23 predictions out of 92, *p*-value of 1.5 x 10^−5^) is the ATP-binding cassette (ABC) family. Its members participate in many biological processes that include vacuolar detoxification, pleiotropic drug resistance (PDR) and stress adaptation (reviewed in [33]). Yap1p participates in the PDR regulation network, and its encoding gene has a verified IRES [34]. Additionally, we predicted four genes containing IRESs in this regulatory network (*PDR5*, *PDR12*, *SNQ2* and *STP5*), two of which have been shown to be directly regulated by Yap1p (*SNQ2* and *PDR5*) [33]. It is important to note that although *YAP1* was not used to train our SVM, it was one of the genes predicted as having an IRES. The above-described case constitutes another example consistent with the hypothesis of multiple IRES-dependent genes in the same regulatory or interaction network [7]. A second example of a gene with an experimentally verified IRES [35–37], that was not used in the training procedure of our SVM, but successfully identified as having an IRES by our method is HAP4. Remarkably, there is functional evidence that HAP4 and YAP1 diverged from a common ancestor [38]. Considering the statistical and biological aspects of the aforementioned predictions, we believe that our results validate our method and support IRES-dependent translation conservation in fungi (Fig. 1).

### Selection of top IRES predictions

The product of the orthologous group enrichment and the complement of the SVM class posterior probability [39] (1-probability that a prediction is an IRES based on SVM output) was used as a criterion for ranking our predictions. We defined a threshold of 0.05 (lower values indicate better predictions) for selecting the best predictions. Our top IRES predictions included only 801 out of the 6,532 total positive predictions (12 %) and represented 0.8 % of the entire set of genes considered in our analysis (99,759). For *S. cerevisiae,* 174 genes out of its nearly 6,000 coding genes were included in the top-predictions category. The advantage of our selection procedure (see Methods) is that it takes into account the similarity of features found in each sequence compared with those of the experimentally verified IRESs used in this study (given by the posterior class probability) and the enrichment of IRES predictions in phylogenetically related genes across organisms, which could indicate a selective pressure to conserve IRES-dependent translational control. All further analyses in our study were performed using this sub-set of 174 top IRES predictions in *S. cerevisiae*.

### Gene ontology enrichment analysis of the predicted IRESs in *S. cerevisiae*

**Fig. 2:**
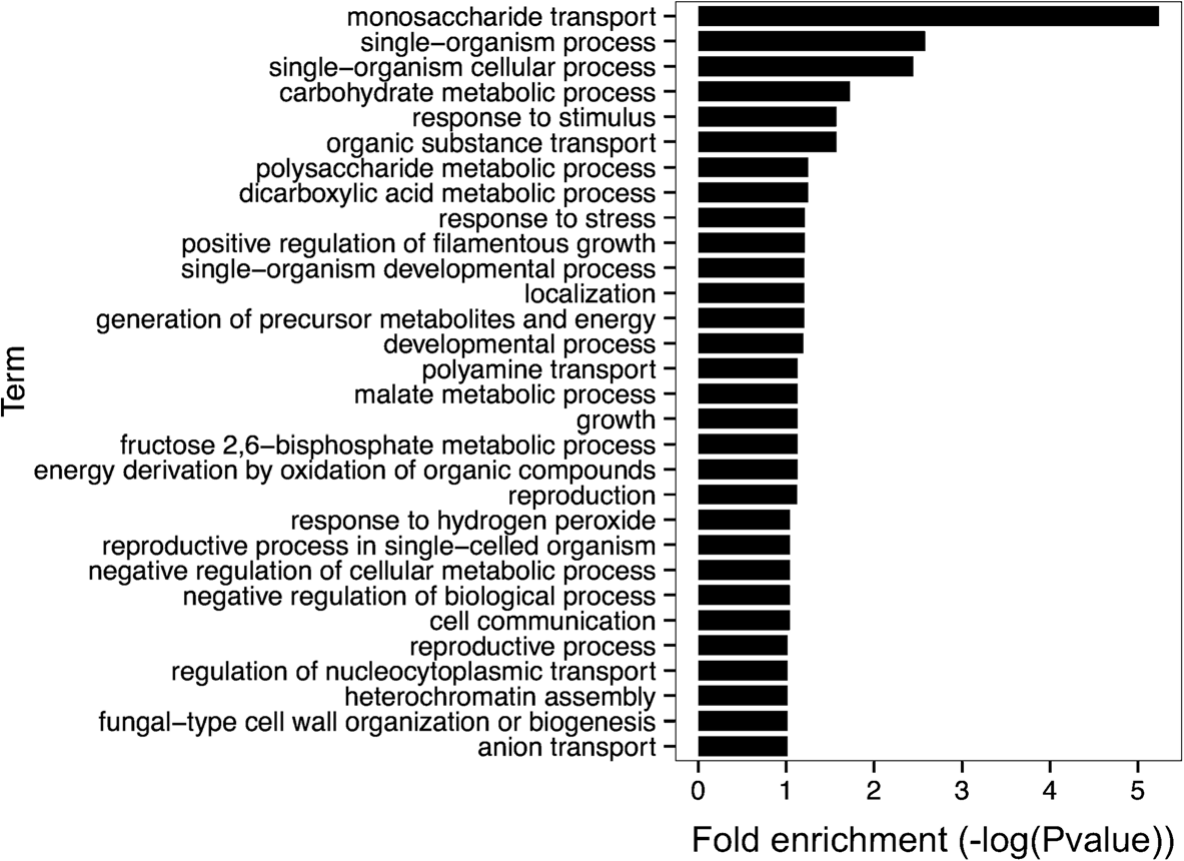
GO-term enrichment analysis for the top IRES predictions. The GO-term enrichment analysis was performed using the GOstats package for R. Fold enrichment values are represented as the minus base 10 log of their corresponding p-values

We performed a gene ontology (GO) [40, 41] enrichment analysis corresponding to “Biological Process (BP)” terms for the top IRES predictions of 174 genes. We found 28 significantly enriched GO terms using FDR adjustment (FDR < 0.1) after summarizing them using REVIGO [42] (Fig. 2). It is worth noting that a number of the enriched GO terms presented here have been associated with 5’-cap-dependent translation suppression and selective protein production through 5’-cap-independent translation, and in several cases, a detailed study of those genes translated in a selective manner led to the discovery of new IRESs. Some of the aforementioned conditions include developmental processes, transport, cell communication [43], filamentous growth [17] and response to stress (reviewed in [7, 44]). As such, these results indicate that our predictions are clearly different from those produced randomly, not only at the phylogenetic level (as shown by orthologous group enrichment) but also at the functional level of proteins participating in specific biological processes.

### Network analysis of the predictions

A functional PPI network comprising the 174 top IRES predictions in *S. cerevisiae* was constructed using data from the STRING database [45]. This database provides information not only for direct physical protein-protein interactions but also considers a broader set of “functional protein-protein associations” comprising participation in common metabolic pathways, co-regulation, and participation in larger structural assemblies [45]. To characterize the PPI network from the perspective of its connectivity, we selected the parameter of network density because it describes the global level of cohesion. Network density is calculated as the ratio of observed connections to possible connections (possible connections refers to the number of links in a fully connected network) [46]. Network density has an intuitive biological meaning in this context because larger network densities are related to higher levels of functional association.

Statistical simulation was used to test how the network density of the PPI network of the top IRES predictions compares with a random network (see Methods). Remarkably, the network density had a value of 0.071, higher than that of the expected value of a random network (0.0461; *p*-value of 1.3 x 10^−4^). These values show that the PPI network built from the top predicted IRESs was more cohesive and significantly different from a random network of the same size.

### IRES-dependent translated proteins are functionally associated into biologically significant modules that participate in specific processes

It has been widely demonstrated that cells perform most of their functions in a modular fashion [47–51]. The prevailing definition of modularity considers a set of proteins connected working together physically or functionally to perform related functions [47, 52]. This definition implies that cellular processes occur via the coordinated action of a number of molecules. Therefore, it is expected that the density of connections in each module will be higher than the density of connections in the entire network because the proteins within each module share a common, relatively homogenous set of functions [48]. In addition, the decomposition of a network into functionally related sub-parts can offer valuable information on how the complete system works, thus facilitating its analysis.

**Fig. 3:**
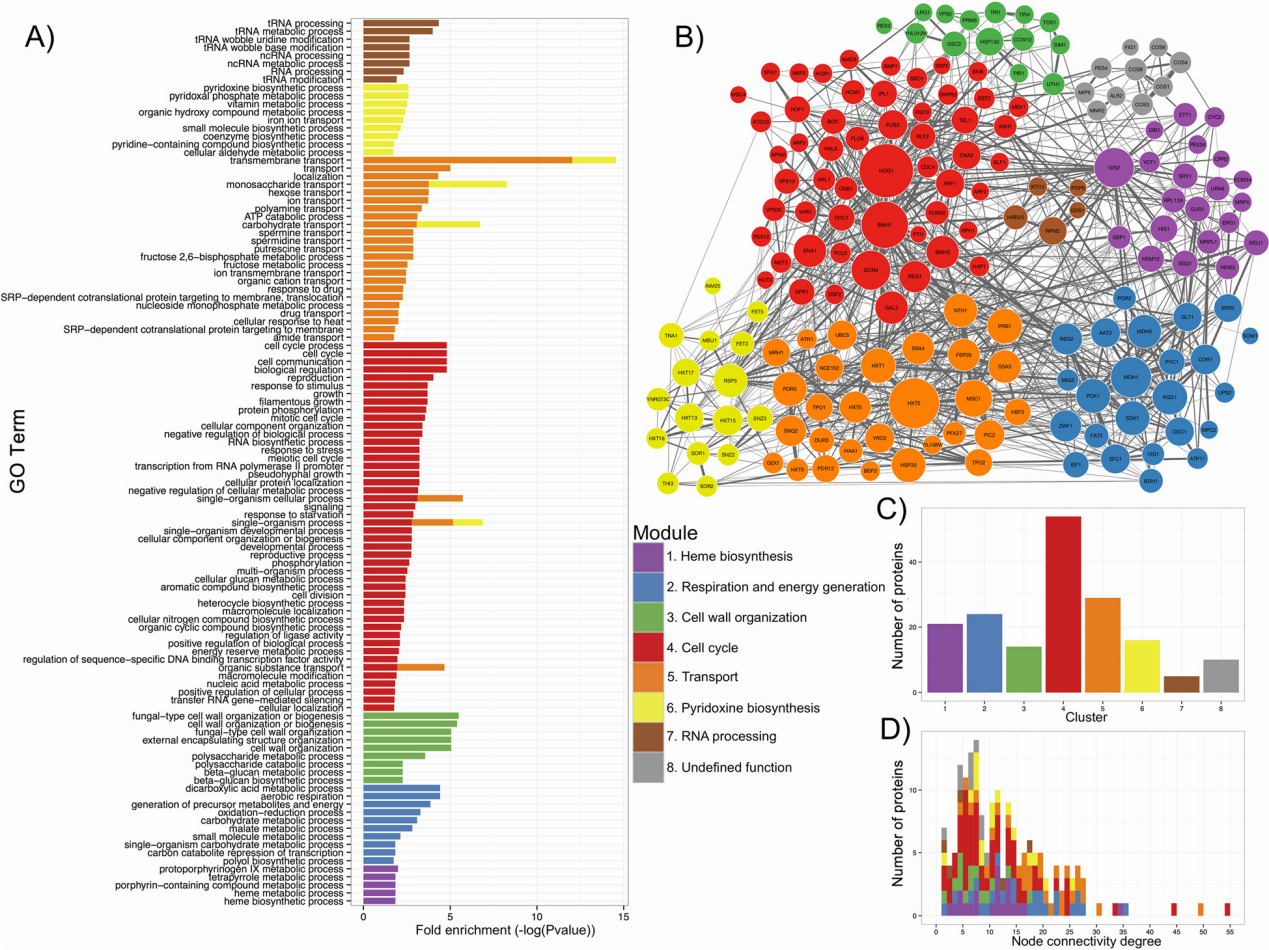
Network clustering of the proteins translated by the set of top IRES predictions in *S. cerevisiae*. Each group is represented by a different color (**a**) GO-term enrichment analysis. The p-values of the GO-term enrichments are represented in logarithmic terms. **b** PPI network. The size of each vertex is proportional to its connectivity degree value. **c** Number of proteins in each module. **d** Node connectivity distribution by module

To characterize the PPI network of the top IRES predictions in terms of its modular structure, we used the Louvain method multi-level unsupervised clustering (module-finding) algorithm [53] implemented in the igraph package [54]. In our study, a total of 9 modules were obtained. One of these modules contained only one protein and was therefore excluded from further evaluations. To explore the biological function of each module, we conducted GO-term enrichment analysis using the same procedure as that applied to the complete set of predictions. This analysis revealed a significant GO-term enrichment (FDR < 0.02) in 7 out of the 8 modules (Fig. 3a). Interestingly, each module exhibited a unique functional specialization because only 6 of the total 117 GO terms were shared (represented by multiple colored bars in Fig. 3a). A closer inspection inside each module also revealed substantial functional homogeneity (Fig. 3a).

The aforementioned results are in good agreement with previous findings of comparable functional module enrichments [55] and support the validity of the clustering procedure used. To facilitate discussion, representative names were assigned to each module based on GO terms: Module 1, heme biosynthesis; Module 2, respiration and energy generation; Module 3, cell wall organization; Module 4, cell cycle; Module 5, transport; Module 6, pyridoxine biosynthesis; Module 7, RNA processing; and Module 8, undefined function, because no GO term enrichment was found.

**Fig. 4:**
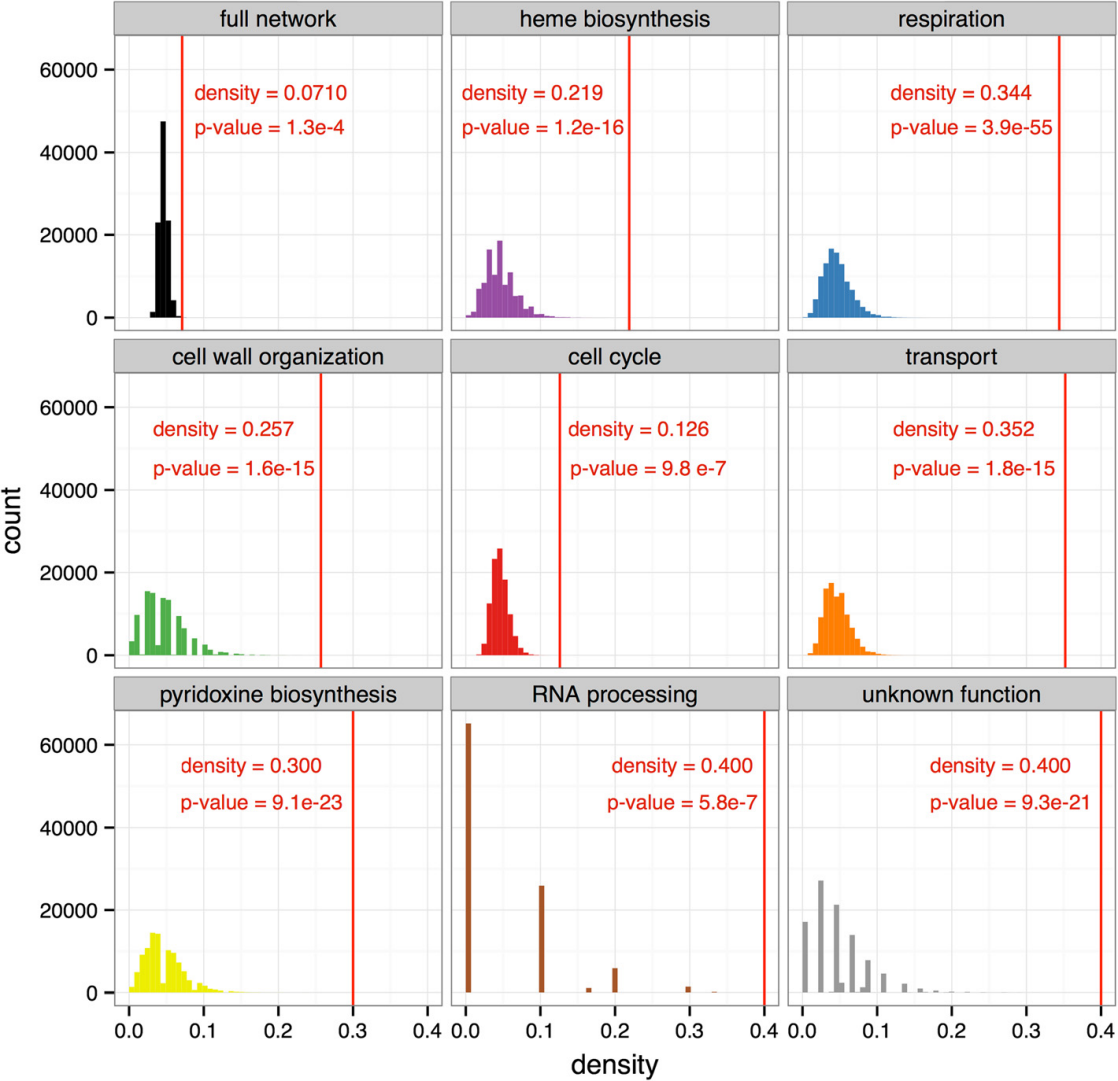
PPI network density of the predicted proteins compared with simulated random networks. The full network and the modules are more cohesive than random networks

In general, higher density values are found when proteins are properly classified into biological modules [55]. For this reason, we compared the modules with the entire network of top IRES predictions in terms of density. Additionally, we compared the density of each module with that of a randomly generated network of a corresponding size (Fig. 4). All modules showed statistically significant higher density values compared with both random networks and the entire network of top IRES predictions. These results are in good agreement with previous studies in which a comparable trend in the density of clusters was observed [55, 56].

We believe the higher density of the modules when compared with either size-equivalent random networks or with the entire network, the GO-term enrichments found in each module, their functional specialization (few shared GO terms between modules), and their functional homogeneity (similar or related functions within a module) strengthen the biological relevance of our IRES predictions. These results support the hypothesis that IRES-dependent translation facilitates the expression of proteins working in a coordinated manner in specific biological processes, such as those previously reported in poliovirus infection, hypoxia and ER stress [7], mitosis [7, 9], apoptosis [10], invasive growth in yeast [17], and the meiotic program [3].

### Biological relevance of the top IRES predictions in the context of their respective modules

In this section, we describe some relevant examples of the top IRES predictions in the biological contexts of their respective modules. We use node connectivity degree (or simply connectivity degree) as a measure of protein importance within the PPI network. The connectivity degree of a given node (protein) represents the number of interactions that this node has in a particular network. The connectivity degree in PPI networks has been linked to the biological significance of proteins. For example, it has been observed that protein connectivity degree is positively correlated with lethality [57] and disease-related genes [58]. The complete list of top IRESs predictions, their module classifications, connectivity degrees and descriptions are presented in Additional file 2.

**Module 1: Heme biosynthesis**. Heme is crucial in many fundamental biological processes and serves as a prosthetic group and a signaling molecule. For example, heme is used in controlling cell growth and differentiation, reducing oxidative damage, generating energy by respiration, and as an enzyme cofactor [59]. In yeast, heme controls transcription in response to oxygen levels through the activator Hap1p [59]. Interestingly, Hap1p is indeed included in the cell-cycle module (module 4), emphasizing the close relationship between the heme group and developmental processes. Additionally, this association suggests a case in which the effector molecule (heme) and the regulated gene (*HAP1*) are translated in an IRES-dependent manner, highlighting the functional association of IRESs.

Another protein with a regulatory function in this module is Gis2p. This protein is the most connected element in this module, with a connectivity degree value of 34. Gis2p is a translational activator of mRNAs with IRESs [60, 61]. Additionally, similar regulatory functions in IRES translation were found for the Gis2p orthologs Znf9p in humans [62] and Cnbp in flies [63], indicating a potentially conserved function. Furthermore, Gis2p is implicated in stress response by its accumulation in P-bodies and stress granules under glucose deprivation conditions [64], and it is part of the genetic network implicated in the induction of invasive growth [65]. Remarkably, seven other genes required for invasive growth are also known to be translated in an IRES-dependent manner in *S. cerevisiae* [17].

The presence of IRES elements in regulatory genes, such as the abovementioned *HAP1* and *GIS2*, implies the existence of a wider hierarchical regulatory network that responds to specific metabolic or stress conditions in which some components of the translation machinery may be depleted.

Another remarkable example of two closely related proteins in this module is Ssq1p, a mitochondrial chaperone of the HSP70 family, and its co-chaperone, Mdj1p [66]. It is worth noting that Ssq1p has two homologs encoded by genes with IRESs, Hsp70p in *D. melanogaster* and in humans [13]. Ssq1p is required for the assembly of iron-sulfur clusters into proteins [67].

An additional example worth noting within this module is Sbp1p. It has two homologous proteins, Pab1p in yeast and Cirp in mice, which are known to be translated in an IRES-dependent manner.

**Module 2: Respiration and energy generation**. The ability to respond to nutrient changes is a crucial requirement for cell survival. *S. cerevisiae* preferentially uses glucose as a carbon source, although in glucose starvation conditions, alternative non-fermentable carbon sources can be used. Two proteins included in this module that belong to the central pathway of gluconeogenesis are malate dehydrogenase (Mdh1p and Mdh2p) and phosphoenolpyruvate carboxykinase (Pck1p). These proteins are degraded in the presence of glucose [68], and their corresponding genes are transcriptionally regulated by the zinc-finger transcription factor Znf1p following glucose starvation [69]. Pck1p also participates in other stress conditions, and it was found to confer cold tolerance in yeast [70].

**Module 3: Cell wall organization**. The cell wall adjusts its thickness and composition to contend with environmental stimuli, such as mechanical, osmotic, and heat shock stresses. The *S. cerevisiae* cell wall is composed largely of polysaccharides (85 %) and proteins (15 %), one of which is predicted to have an IRES: Gsc2p, a β-1,3-glucan synthase that can be induced by different environmental stimuli. For example, extracellular calcium, treatment with α-factor [71], heat shock [72], exposure to cell wall-damaging agents [73], or treatment with the reducing agent dithiothreitol [74] leads to strong Gsc2p induction.

The cell wall adapts its shape during different developmental and growth stages (reviewed in [75]). For example, the products of *HSP150*/*PIR2* and *PIR1*, which are predicted to contain IRES sequences, are required for cell wall stability [76], but how these proteins contribute is still unclear. However, PIR proteins are known to impact the permeability of the cell wall, and this effect is consistent with the role of these proteins in cross-linking β-1,3-glucans [77, 78].

Hsp150p and Pir1p are induced by heat shock, treatment with CFW or Zymolyase, and nitrogen limitation [77, 79, 80]. They are also regulated during cell cycle progression and in response to stress [81]. Additionally, there is evidence of coordinated regulation between genes required for cell wall organization or biogenesis and cell cycle genes [82]. Importantly, evidence has been found for translational control through 5’-UTRs in the case of two of the proteins in this module, Uth1p and Sim1p [83].

**Module 4: Cell cycle.** Module 4 is the largest module in terms of the number of enriched GO terms (Fig. 3a) and the number of genes (Fig. 3c). Additionally, this module includes the nodes displaying the highest connectivity degrees.

Importantly, the translational regulation of cell cycle processes has been described in several studies, and a number of IRESs play central roles regulating the expression of different kinases (reviewed in [9]).

The most connected protein in module 4 is Hog1p (High Osmolarity Glycerol response), which has a connectivity degree of 54 (Fig. 3c). Remarkably, Hog1p has 3 homologous proteins in humans that are translated in an IRES-dependent manner (PITSLREp, Pim1p and calcium/calmodulin-dependent protein kinase type II subunit alpha) [13]. This finding supports IRES conservation even in distant organisms. Hog1p is a mitogen-activated protein kinase that has important roles in different stress conditions. For example, the translational response to hyperosmotic shock is strongly dependent on Hog1p [24], and this protein has been shown to control cell cycle progression in response to stress [84]. It is worth noting that other kinases are part of this module and have important regulatory roles in the cell cycle (Tel1p [85], Ipl1p [86], Vhs1p [87], and Mek1p [88]), cell growth and proliferation (Cka2p [89]), salt tolerance (Hal5p [90]), and mating (Fus3p [91]).

Another relevant protein in this module is Gcn4p, which is a master translation factor that activates the response to amino acid starvation [92] and is controlled at both the transcriptional and translational levels by diverse signals of stress [93]. Within the network of top IRES predictions, Gcn4p has a high connectivity degree (33), possibly reflecting its importance. Significantly, the Gcn4p paralog Yap1p and the ortholog Jun protein in chicken are encoded by genes with experimentally verified IRESs [13].

A final example of a remarkably significant protein in this module is Med10p (Nut2p), a subunit of the RNA II mediator complex that is required for transcriptional activation because its concentration is elevated in response to DNA replication stress [94, 95]. In addition, the mRNAs of two other proteins that are part of this complex, Med7p and Med18p (Srb5p), were predicted to be encoded by genes with IRESs. This finding has biological relevance because several components of the same complex are translated in an IRES-dependent manner, providing selective co-regulation under stress conditions. Additionally, there is evidence of the co-regulation of some proteins in these complexes at both the transcriptional and translational levels [96–98].

**Module 5: Transport.** The biological relevance of some members of this module has already been discussed because they are part of the most enriched orthologous groups (*HAA1*, *HXT1*, *HXT5*, *HXT6*, *HXT9*, *PDR5*, *PDR12*, *PDR15*, *SSA4*, and *SNQ2*; see *Enrichment analysis of IRES predictions in Orthologous Groups*). The proteins in this module are involved in the transport of a wide range of molecules. For example, Ssa3p and Ssa4p participate in SRP-dependent co-translational protein-membrane targeting and translocation [99], HXT members sense and transport glucose [23], Snq2p is an ABC transporter that confers multidrug resistance [100], Tpo1p and Tpo2p function as polyamine transporters [101], Yro2p is a plasma membrane protein involved in resistance to weak acid stress [102, 103], and Haa1p is a transcriptional activator that regulates *TPO2* and *YRO2* [104]. In the context of stress response, transport mechanisms play fundamental roles. For example, multiple transporters are involved in the response to weak acid stress, including the aforementioned Snq2p, Tpo1p, Tpo2p, Pdr12p, etc. [105]. Other stress conditions relevant to the members of this module are osmotic stress, oxidative stress, heat shock and detoxification [33] (see Additional file 2).

**Module 6: Pyridoxine biosynthesis (VitB6).** The most studied role of VitB6 is as a cofactor of enzymatic reactions. However, it is now clear that VitB6 is a potent antioxidant that protects cells from oxidative stress [106, 107]. Moreover, Snz2p and Snz3p, which are part of this module, are known to respond to nutrient limitation [108].

**Module 7: RNA processing.** This module exhibited an enrichment of terms related to RNA processing, including ncRNA and tRNA. Although there are no IRESs known to be associated with RNA processing, we believe that the predicted genes presented in this study are plausible because RNA-based regulation is an area that we are just beginning to understand [109], and our findings may have direct implications in stress response and pathological processes [110]. It is worth noting that the protein Hrr25p, which is a part of this module and is involved in tRNA wobble uridine modification [111], is a likely homolog of the Pim-1 protein, which has an experimentally verified IRES [13].

**Module 8: Undefined function**. Although module 8 was not enriched with specific GO terms, 5 out of the 10 members of this module were COnserved Sequence (COS) proteins (Cos1p, Cos3p, Cos4p, Cos6p and Cos8p), which are highly conserved in sequence, although their functions are unknown [112].

Interestingly, two RNA-binding paralogs, Pes4p and Mip6p, are included in this module. Both proteins are also homologous to Pap1p, the translation of which is IRES-dependent and part of the translation initiation complex [17], providing evidence of selective IRES conservation in homologous genes.

We believe that the fact that our IRES predictions clustered in cohesive GO-enriched modules highlights the functional association of IRES-translated genes and is complementary with our comparative genomics and network analyses. Furthermore, our modular organization-based approach could be used to analyze the results from genome-wide studies addressing IRES-dependent translation.

### Comparison of predictions with translationally regulated genes

Ribosome profiling is a recently developed technique that has contributed to the understanding of the translation process by enabling the determination of the positions and dynamics of active ribosomes along the message, allowing the identification of translationally controlled genes. For this reason, we selected a previously published study [3] based on ribosome profiling to determine the intersection of our predictions and those genes that were found to be translationally controlled. Selected genes had the additional characteristic that their 5’-UTR ribosome occupancy rates were positively correlated with their translation efficiencies, indicating that augmented protein production is a consequence of increased ribosome occupancy. The number of translationally regulated *S. cerevisiae* genes in the aforementioned study was 110, whereas the number of top *S. cerevisiae* IRES predictions was 174; the intersection between these two sets of genes is 14 genes. The probability of having an intersection of this size at random considering 6,000 coding genes in *S. cerevisiae* is 5 x 10^−7^ (Fisher’s exact test), which is a good indication of the accuracy of our predictions. Representative examples of ribosome footprints for genes with experimentally verified IRESs, genes predicted as having IRES and genes predicted as not having IRESs, are presented in Additional file 3. Data obtained from reference [2].

## Conclusions

We developed an accurate computational method based on a SVM for the identification of unstructured A-rich IRESs in fungal organisms. Using this method, we predicted IRES elements in the 5’-UTR sequences of 20 non-redundant fungal genomes and performed a comparative analysis and characterization of the functional relationships among the proteins encoded by the genes predicted to have IRES elements. We found statistically significant conservation of IRES-dependent translation in some groups of orthologous genes that revealed an underlying selective pressure, particularly in stress-related genes. In addition, our network analyses allowed us to identify biologically meaningful modules exhibiting specialized functions, providing evidence of a strong functional association between IRES-dependent translated proteins. Our study represents a useful resource for hypothesis-driven experiments and gene function exploration in the field of cap-independent translational regulation.

## Methods

### DNA sequence data

In this study we used sequence and annotation data from 33 completely sequenced fungal genomes. To avoid data overrepresentation, non-redundant genomes were selected based on their position in a maximum likelihood phylogenetic tree that was constructed using the PHANGORN package [113] available in the R software [114]. For each pair of phylogenetically close organisms, the one with the smaller genome was eliminated, leaving the organism with the larger genome [115]. The final set of non-redundant organisms used in our analysis consisted of 20 organisms. The complete list of organisms used, the list of non-redundant organisms and the phylogenetic tree are provided in Additional files 4, 5 and 6, respectively.

### Obtaining the 5’-UTR sequences in this study

It has been shown that several yeast IRESs are located within the region corresponding to the first 60 nt immediately upstream of the translation initiation codon [17, 18]. Consequently, using a Perl script, the aforementioned region was obtained for each gene in each of our non-redundant yeast genomes. We termed these sequences 60ntUTRs, and they were used in our subsequent analyses. These 60ntUTRs were sorted in accordance with the orthologous groups of their corresponding genes. In *S. cerevisiae*, as far as we know, there are 11 experimentally confirmed and well-characterized IRESs with the common characteristic of being A-rich sequences [17, 18], 9 of these constitute the positive cases for the training of our SVM, whilst 2 of them were used as our internal positive control. The list of these genes and their characteristics are given in Additional file 7. Negative cases were obtained by the random sampling of 12,500 sequences from the complete pool of 60ntUTRs. This number of negative cases was selected because when compared with the number of positive cases generated by SMOTE (5,000) gives a ratio of 2.5:1. It should be noted that it was not easy to select cases that represent a truly 100 % negative control supported by experimental studies. Nevertheless, based on microarray analyses it has been estimated that only 10-15 % of mRNAs remain attached to polyribosomes under different stress conditions and considering that only 4 % of them might exhibit cap-independent translation [43]; we estimated that only 4-6 % of the genes used in the negative set for the training of our SVM might contain an IRES.

### Feature selection for the prediction of IRES elements

Feature selection of the 60ntUTRs to predict IRES elements was based on a literature review, considering those variables that have been reported as correlated with the presence of IRESs or those that have been used to classify non-coding RNA (ncRNA). The set of features used in our analysis was grouped according to the following criteria: i) Minimum folding energy (MFE), which has been used to classify non-coding RNAs (ncRNA) [116] and is correlated with IRES expression strength in yeast [18]. The MFE of each of the 60ntUTRs was calculated using the RNAfold program of the Vienna RNA package, version 1.8.5 [117]. ii) GC content. It has been proposed that IRES-possessing *S. cerevisiae* genes related to nitrogen starvation tend to be A-rich [17]. Additionally, a positive correlation has been observed between the low GC content of UTRs and increased translational activity in glucose starvation conditions, which might be explained, at least partially, by IRES elements promoting protein expression [118]. For these reasons, two features were included: the GC content of the 60ntUTRs relative to the GC content of their corresponding intergenic regions (relGCintergenic) and the GC content of the 60ntURs relative to the chromosomal GC content (relGCchr). iii) Relative gene position in the chromosome (relPosChr). This feature was used because it has been shown to be related to the selective expression of stress-response genes [28, 119–121]. iv) Di-nucleotide frequencies. Composition-based approaches have been successfully used to develop ncRNA classification methods [122]. Therefore, we calculated di-nucleotide frequencies (16 variables) as input features. v) Measures of statistical dispersion. The higher the conservation of IRESs in an orthologous group, the more influence these elements will have on the properties of their group. For this reason, the mean, mode, standard deviation, and skewness of each of the ortholog groups were calculated for the relGCintergenic and the MFE of its sequence members. vi) Length of the intergenic region. This feature was selected for IRES identification because longer regions could be indicative of the presence of regulatory elements, such as IRESs. After applying the criteria described above, a total of 29 features were used as inputs in our SVM. The lists of features before and after selection are provided in Additional file 1. In order to have a relative estimation of the likely contribution of these features in our SVM, we compared the average values of the features of the positive predictions *versus* the average values of the negative set of sequences used to train our SVM. The result of these comparisons is show in the figure of Additional file 8. As it was expected, in this figure, the *AA* and the *GC dinucleotides* presented the most significant values. Other features with important coefficient values were the *intergenic region length* and those features related with the minimum folding energy of the 60ntUTRs.

### Feature pre-processing

To render the inter-species attributes comparable, feature standardization was performed to rescale the variables by their means and variances relative to their distributions in each organism. Subsequently, all the features had a mean of 0 and a variance of 1. To avoid data redundancy, we reduced the number of features to retain only uncorrelated variables [123]. A pair of features exhibiting a Pearson correlation factor greater than 0.55 were considered to be correlated. For each binary combination of correlated features, one was eliminated. After this step, 25 features from the original set of 29 were retained (see Additional file 1).

### Synthetic minority oversampling

In general, machine learning methods perform poorly when they are applied to imbalanced datasets in which negative cases heavily outnumber positive cases [124]. However, in real data sets imbalances ranging from 100:1 up to 10,000:1 have been reported [125]. This type of datasets are common in biological studies as well, for example, in the prediction of translation initiation sites [126] and pre-miRNA classification [127]. The dataset used in this work is imbalanced because the number of A-rich IRESs in *S. cerevisiae* used in the training of our SVM (9 sequences) is very small compared to all possible genes containing IRESs (nearly 100,000 for the 20 selected organisms). To address this imbalance, synthetic minority oversampling technique, SMOTE [124] implemented in the DMwR package [128], was used. SMOTE is an oversampling technique that generates synthetic minority class samples by randomly choosing elements along the line segments joining some of the k minority class nearest neighbors [124]. This technique was selected because it has been shown to significantly improve the performance of SVMs used to classify non-coding RNAs (ncRNAs) [127], to predict RNA-protein interactions [129], and to analyze other imbalanced bioinformatic datasets [130] when this imbalance is superior to 100:1 [129, 131, 132]. It is worth mentioning that in our analysis, this imbalance was more significant than those previously reported (nearly 10,000:1). Additionally, considering the limited number of positive cases, this represents a potential constraint for the generalization of our predictions outside the training set. Considering this concern, our study includes two external positive cases of experimentally confirmed IRES not used in the training procedure (see Methods section). Furthermore, we performed an extensive and detailed statistic analysis of the enrichment of our IRES prediction in specific orthologous and functional groups (PPI network analysis) that supports the validity and generalization capacity of our IRES identification method (see Results and discussion section). For the SMOTE procedure, the parameter k was set to 300, the over-sampling to 900, and the under-sampling to 500, resulting in a subset of 17,500 genes.

### Machine learning for IRES prediction

A support vector machine with a second-order polynomial kernel implemented in the caret package [133] was used for training on the selected features for IRES prediction. To increase the sensitivity, a cost of 2:1 (positive prediction: negative prediction) was set for the SVM [134]. Cross-validation (10-fold) repeated 30 times was used to measure the performance of the SVM. Predictions were evaluated using the set of 100,000 genes. Posterior class probabilities P(class|input) were calculated for each prediction according to Platt’s methodology [39]. The complete list of predictions is provided in Additional file 9.

### Enrichment analysis for the predictions

Genes predicted to contain IRESs were assigned to their corresponding orthologous groups. Fisher’s exact test was performed to determine enrichment significance [19], and the resulting *p*-values were corrected for multiple testing using the Benjamini-Hochberg procedure [135].

### Comparison of predictions with experimental data

To compare the probability of a random intersection of the IRES predictions with sets of translationally-controlled genes determined experimentally [3], Fisher’s exact test was used [19].

### Gene ontology analysis

The GO enrichment analysis [40, 41] was performed using the GOstats package [136] in the R software [114], correcting for multiple comparisons via the Benjamini-Hochberg method [135].

### Selection of top IRES predictions

To select the best IRES predictions, we used a simple procedure that consisted of multiplying the posterior class probability [39] (the probability that a prediction is an IRES based on SVM output) of each prediction by its corresponding orthologous group enrichment *p*-value. We ranked the predictions according to this product (lower values indicate better predictions) and established a cutoff of 0.05. We used only genes classified as having IRESs by the SVM to avoid increasing the misclassification. The list of top predictions can be found in Additional file 2.

### Protein-protein network analysis

Interaction data were obtained from the STRING database version 9.1 [45] using the STRINGdb package in the R software [45]. Graph properties were calculated using the Louvain method [53] implemented in the igraph package [54]. The corresponding number of genes in the complete network or in the particular module (Fig. 3c) to be evaluated was sampled at random from the entire list of protein-coding genes in *S. cerevisiae.* Afterwards, a network was constructed using the sampled genes, and its graph properties were calculated. This process was repeated 100,000 times for each case. The data were Box-Cox transformed to approximate normal distributions [137]. The normal distribution function was applied to calculate the *p*-values using the R software [114].

### Protein homology determination

In order to determine if two proteins are homologous, pairwise comparison was performed using delta-blast [138], with an e-value threshold of 1 x 10^−6^ .

### Availability of supporting data

DNA sequences and annotations were obtained from the Entrez Genome Database (ftp://ftp.ncbi.nlm.nih.gov/genomes/) [139]. The complete list of organisms and their corresponding accession numbers are in Additional file 4. The R software is available from https://www.r-project.org/. Groups of orthologous genes were downloaded from ftp://cegg.unige.ch/OrthoDB7/OrthoDB7_ALL_FUNGI_tabtext.gz [140]. The data of genes having IRESs was obtained from http://iresite.org/ [13] and from [17], and its respective supplement: http://www.sciencemag.org/content/317/5842/1224/suppl/DC1.

The list of translationally controlled genes determined by ribosome profiling was downloaded from the materials and methods supplement of [3]: http://www.sciencemag.org/content/335/6068/552/suppl/DC1. All the programs used in our analysis are available at our web page http://www.ibt.unam.mx/biocomputo/IRES_programs.html or at the figshare website http://dx.doi.org/10.6084/m9.figshare.1598203.

## Additional files

Additional file 1:
**Features used to train our SVM**. The initial 29 features to train our SVM are listed. After the selection of non-correlated features, 25 features from the original set of 29 were retained. A pair of features exhibiting a Pearson correlation factor greater than 0.55 were considered to be correlated. (XLS 21 kb) Additional file 2:
**Top predictions.** List of top genes predicted as having IRESs, including the names of the genes, their GI identifiers, their corresponding modules (clusters) in the PPI network, short descriptions/aliases, and long descriptions. (XLS 98 kb) Additional file 3:
**Comparison of ribosome profiles.** Ribosome profiles for *Saccharomyces cerevisiae* grown in rich media and under starvation conditions are presented. A) and B) are two genes that have verified IRESs. C) and D) are positive predictions and E), and F) are negative predictions. The regions presented comprise the 60 nt upstream and the 100 nt downstream the start codon. The translation initiation site is shown (blue vertical lines). The genes having IRESs present an increment ribosomes occupancy within the 60 nt upstream the translation initiation in starvation conditions compared to rich media. The two positive predictions depicted similar increments. These increments are not observed in the two negative predictions. The profiles were obtained using the web page http://gwips.ucc.ie/. (PNG 980 kb) Additional file 4:
**List of organisms initially considered for the analysis**. (XLS 29 kb) Additional file 5:
**List of non-redundant organisms in the analysis.** Non-redundant genomes were selected based on their position in a maximum likelihood phylogenetic tree that was constructed using the PHANGORN package [113] available in the R software (http://www.r-project.org) [114]. (XLS 21 kb) Additional file 6:
**Phylogenetic tree.** Phylogenetic tree used to select the non-redundant organisms. Bootstrap supporting values are indicated. (PNG 79 kb) Additional file 7:
**Description of experimentally verified IRESs in** ***Saccharomyces cerevisiae*** **.** A table containing their sequences, description and references. (XLS 34 kb) Additional file 8:
**Comparison of the feature averages of the IRES predictions to the negative set used for training.** The y-axis represents the log2 ratio of the feature averages for the positive predictions to the negative set. Higher values for the positive predictions are depicted in red and lower values in green. The Wilcoxon rank sum test was used to test the differences between means comparing the positive and negative sets. (PNG 88 kb) Additional file 9:
**Complete predictions.** List of genes predicted to have IRESs, including the GI identifier, gene name, orthologous group, posterior probability of being an IRES, orthologous group enrichment, product of the posterior probability and group enrichment, and the mRNA sequence used. (XLS 1571 kb)

## Abbreviations

60ntURs: First 60 nt immediately upstream of the translation initiation codon
ABC: ATP-binding cassette
BP: Biological Process
COS: Conserved sequence
FDR: False discovery rate
GO: Gene ontology
IRES: Internal ribosome entry sites
MFE: Minimum folding energy
ncRNA: non-coding RNAs
nt: Nucleotide
OG: Orthologous group
PDR: Pleiotropic drug resistance
PPI: Protein-protein interaction
relGCchr: Relative to the chromosomal GC content
relGCintergenic: Relative to the GC content of their corresponding intergenic regions
relPosChr: Relative gene position in the chromosome
SVM: Support vector machine
