# Supplementary figures and images for: IRES-dependent translated genes in fungi: computational prediction, phylogenetic conservation and functional association
Source: BMC Genomics. 2015 Dec 15;16:1059. doi: 10.1186/s12864-015-2266-x (PMC4678720; doi:10.1186/s12864-015-2266-x)

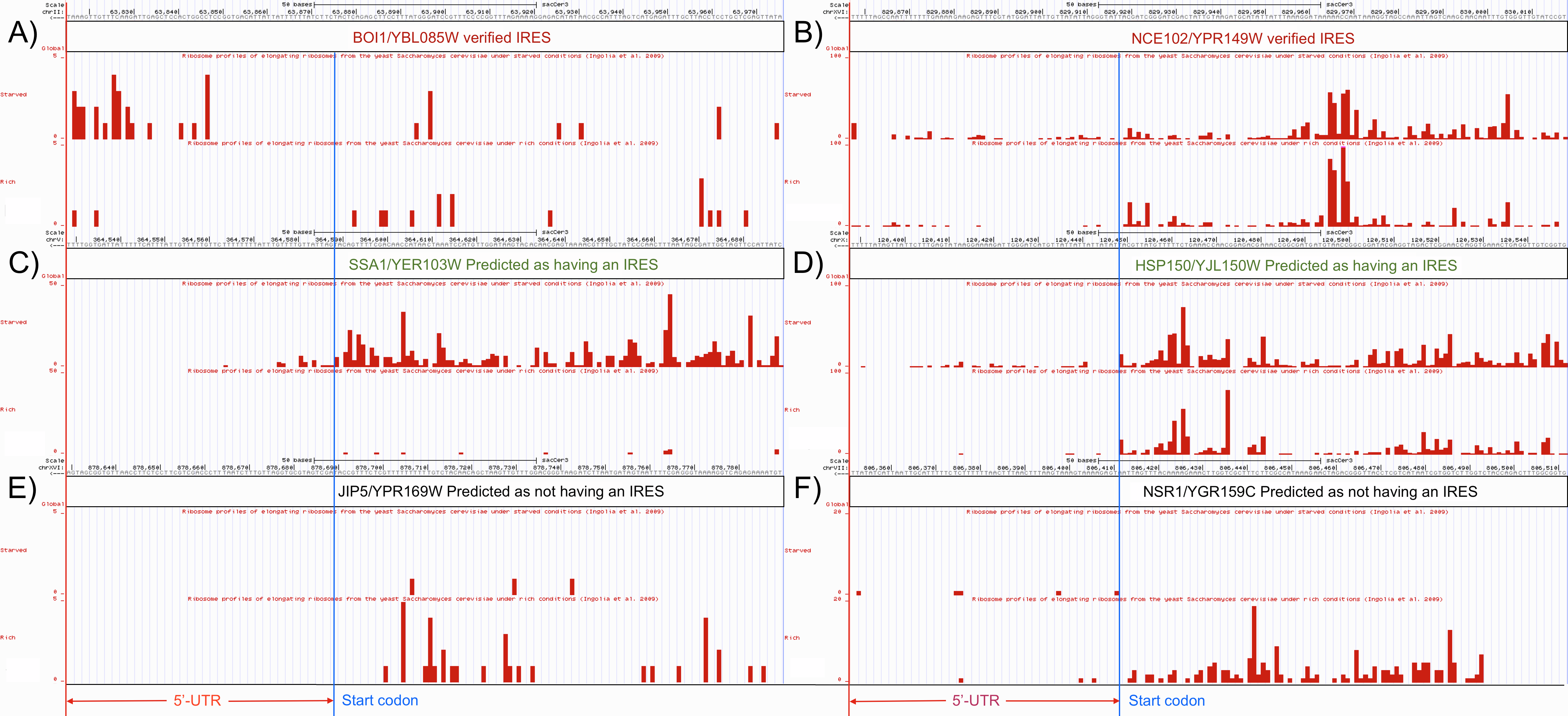

Supplement: Additional file 1: — Features used to train our SVM. The initial 29 features to train our SVM are listed. After the selection of non-correlated features, 25 features from the original set of 29 were retained. A pair of features exhibiting a Pearson correlation factor greater than 0.55 were considered to be correlated. (XLS 21 kb) [file 12864_2015_2266_MOESM1_ESM.png]

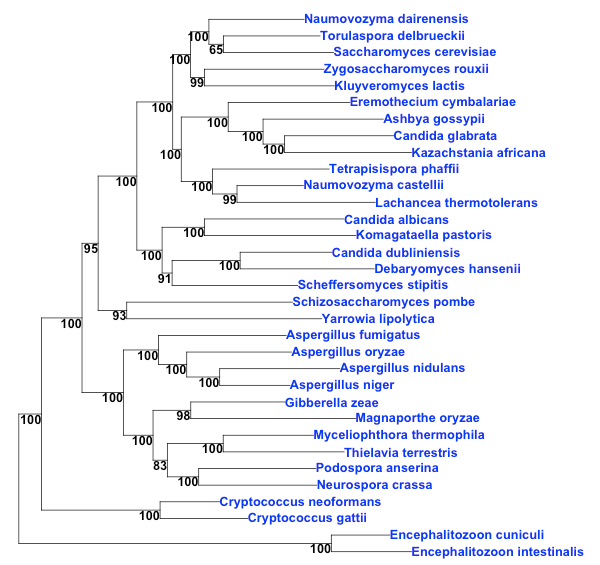

Supplement: Additional file 4: — List of organisms initially considered for the analysis. (XLS 29 kb) [file 12864_2015_2266_MOESM4_ESM.png]

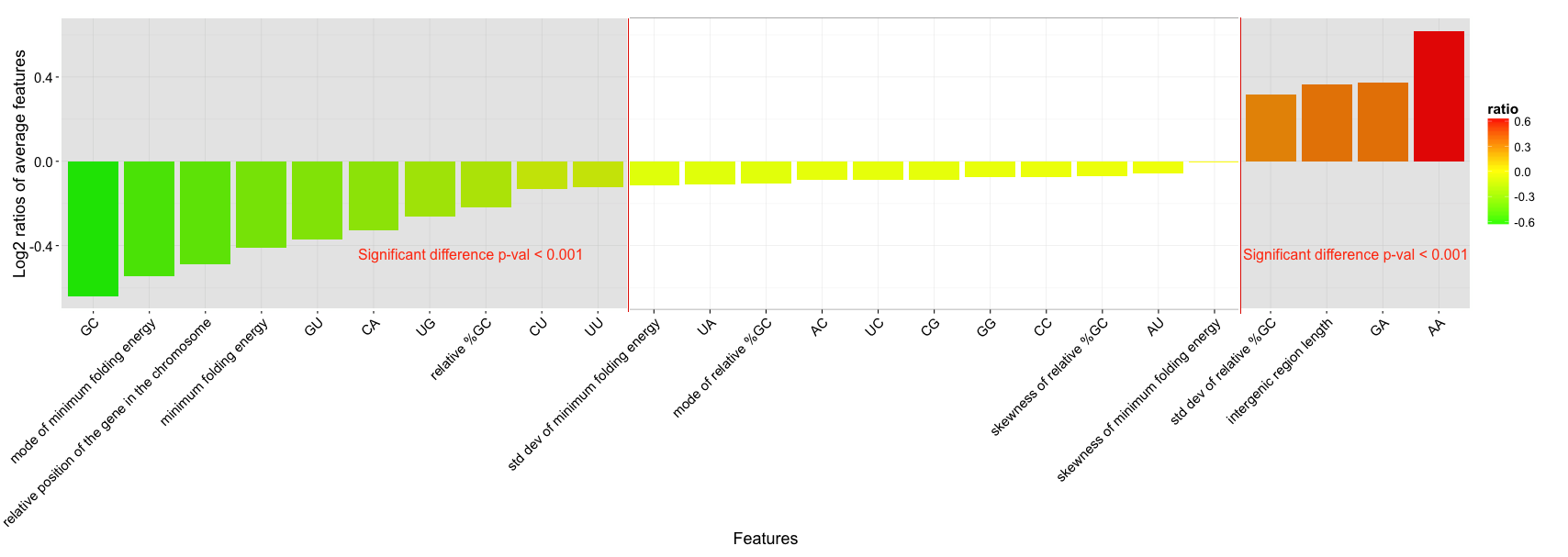

Supplement: Additional file 7: — Description of experimentally verified IRESs in Saccharomyces cerevisiae . A table containing their sequences, description and references. (XLS 34 kb) [file 12864_2015_2266_MOESM7_ESM.png]
